# The quality of EDIFACT referrals from primary care to the emergency department

**DOI:** 10.1186/1757-7241-21-S2-A46

**Published:** 2013-09-09

**Authors:** Hanne Hestbech, Søren W Hansen, Thomas A Schmidt

**Affiliations:** 1 The Emergency Department, Holbaek, University Hospital, Denmark

## Background

Communication between primary care and the Emergency Department is in most cases provided through written referrals. The quality is very important to create coherent clinical courses, treatment of high quality and high satisfaction among patients. 95% of all admissions from primary care are delivered as EDIFACT, which is a secure way to send electronic post and to ensure immediate delivery. There’s already a guideline that describes the requirements to EDIFACT admissions but no studies describe the quality and the use of them.

## Methods

A retrospective study that included 228 patients admitted to the Emergency Department from primary care. We registered all admitted patients on 10 randomly selected weekdays in the period from the 1st of October to the 15th of December 2012. Only patients admitted from primary care via EDIFACT were included. All admissions have been thoroughly read and the content compared with the guideline.

## Results

419 patients were admitted to the Emergency Department on 10 randomly selected days. Of these 419 patients 228 were admitted from primary care but only 140 (61%) were referred by EDIFACT. All referrals (100%) contained medical history, 16 admissions (11%) contained information about allergies, 44 admissions (31%) contained information about the patient’s medication and 32 admissions (23%) contained information about vital parametres.

## Conclusion

It may be concluded that the quality of EDIFACT referrals from primary care varies a lot, but we also registered that the guideline isn’t available for doctors in primary care. This isn’t appropriate because important information about admitted patients is lost which results in poorer clinical courses. The 1st of January 2013 new and better guidelines are published which is a good possibility to clarify the importance of EDIFACT referrals. Because of time pressure in primary care one might consider the possibility of developing a new program in which information about the patient’s medication and allergies are captured directly from the primary care charts. This might ensure that important information isn’t lost when doctors in primary care are communicating with the Emergency Department.

